# Supplementary material for: Resolving the full spectrum of human genome variation using Linked-Reads
Source: Genome Res. 2019 Apr;29(4):635–45. doi: 10.1101/gr.234443.118 (PMC6442396; doi:10.1101/gr.234443.118)
Supplement: Supplemental Material [file supp_gr.234443.118_Supplemental_longranger-2.2.zip › longranger-2.2.tar.bz2!/lr2.2-public/lib/stan/cmdstan/stan/doc/api/html/multi__normal_8hpp_source.html]

Stan: src/stan/prob/distributions/multivariate/continuous/multi\_normal.hpp Source File


|  |  |
| --- | --- |
|  | Stan  2.5.0  probability, sampling & optimization |


- Main Page
- Related Pages
- Namespaces
- Classes
- Files

- File List
- File Members

All Classes Namespaces Files Functions Variables Typedefs Enumerations Enumerator Friends Macros Pages

- src
- stan
- prob
- distributions
- multivariate
- continuous

multi\_normal.hpp

Go to the documentation of this file.

1 #ifndef STAN\_\_PROB\_\_DISTRIBUTIONS\_\_MULTIVARIATE\_\_CONTINUOUS\_\_MULTI\_NORMAL\_HPP

2 #define STAN\_\_PROB\_\_DISTRIBUTIONS\_\_MULTIVARIATE\_\_CONTINUOUS\_\_MULTI\_NORMAL\_HPP

3

4 #include <boost/random/normal\_distribution.hpp>

5 #include <boost/random/variate\_generator.hpp>

6

7 #include <stan/agrad/rev.hpp>

8 #include <stan/agrad/rev/matrix.hpp>

9 #include <stan/math/error\_handling.hpp>

10 #include <stan/math/matrix/trace\_inv\_quad\_form\_ldlt.hpp>

11 #include <stan/math/matrix/log\_determinant\_ldlt.hpp>

12 #include <stan/meta/traits.hpp>

13 #include <stan/prob/constants.hpp>

14 #include <stan/prob/traits.hpp>

15 #include <stan/math/error\_handling/matrix/check\_ldlt\_factor.hpp>

16 #include <stan/math/error\_handling/matrix/check\_size\_match.hpp>

17 #include <stan/math/error\_handling/check\_finite.hpp>

18 #include <stan/math/error\_handling/matrix/check\_symmetric.hpp>

19

20 namespace stan {

21

22  namespace prob {

23

24  template <bool propto,

25  typename T\_y, typename T\_loc, typename T\_covar>

26  typename boost::math::tools::promote\_args<typename scalar\_type<T\_y>::type, typename scalar\_type<T\_loc>::type, T\_covar>::type

27  multi\_normal\_log(const T\_y& y,

28  const T\_loc& mu,

29  const Eigen::Matrix<T\_covar,Eigen::Dynamic,Eigen::Dynamic>& Sigma) {

30  static const char\* function = "stan::prob::multi\_normal\_log(%1%)";

31  typedef typename boost::math::tools::promote\_args<typename scalar\_type<T\_y>::type, typename scalar\_type<T\_loc>::type, T\_covar>::type lp\_type;

32  lp\_type lp(0.0);

33

34  using stan::math::check\_size\_match;

35  using stan::math::check\_finite;

36  using stan::math::check\_not\_nan;

37  using stan::math::check\_positive;

38  using stan::math::check\_symmetric;

39  using stan::math::check\_ldlt\_factor;

40

41  check\_size\_match(function,

42  Sigma.rows(), "Rows of covariance parameter",

43  Sigma.cols(), "columns of covariance parameter",

44  &lp);

45  check\_positive(function, Sigma.rows(), "Covariance matrix rows", &lp);

46  check\_symmetric(function, Sigma, "Covariance matrix", &lp);

47

48  stan::math::LDLT\_factor<T\_covar,Eigen::Dynamic,Eigen::Dynamic> ldlt\_Sigma(Sigma);

49  check\_ldlt\_factor(function,ldlt\_Sigma,"LDLT\_Factor of covariance parameter",&lp);

50

51  VectorViewMvt<const T\_y> y\_vec(y);

52  VectorViewMvt<const T\_loc> mu\_vec(mu);

53  //size of std::vector of Eigen vectors

54  size\_t size\_vec = max\_size\_mvt(y, mu);

55

56  //Check if every vector of the array has the same size

57  int size\_y = y\_vec[0].size();

58  int size\_mu = mu\_vec[0].size();

59  if (size\_vec > 1) {

60  int size\_y\_old = size\_y;

61  int size\_y\_new;

62  for (size\_t i = 1, size\_ = length\_mvt(y); i < size\_; i++) {

63  int size\_y\_new = y\_vec[i].size();

64  check\_size\_match(function,

65  size\_y\_new, "Size of one of the vectors of the random variable",

66  size\_y\_old, "Size of another vector of the random variable",

67  &lp);

68  size\_y\_old = size\_y\_new;

69  }

70  int size\_mu\_old = size\_mu;

71  int size\_mu\_new;

72  for (size\_t i = 1, size\_ = length\_mvt(mu); i < size\_; i++) {

73  int size\_mu\_new = mu\_vec[i].size();

74  check\_size\_match(function,

75  size\_mu\_new, "Size of one of the vectors of the location variable",

76  size\_mu\_old, "Size of another vector of the location variable",

77  &lp);

78  size\_mu\_old = size\_mu\_new;

79  }

80  (void) size\_y\_old;

81  (void) size\_y\_new;

82  (void) size\_mu\_old;

83  (void) size\_mu\_new;

84  }

85

86  check\_size\_match(function,

87  size\_y, "Size of random variable",

88  size\_mu, "size of location parameter",

89  &lp);

90  check\_size\_match(function,

91  size\_y, "Size of random variable",

92  Sigma.rows(), "rows of covariance parameter",

93  &lp);

94  check\_size\_match(function,

95  size\_y, "Size of random variable",

96  Sigma.cols(), "columns of covariance parameter",

97  &lp);

98

99  for (size\_t i = 0; i < size\_vec; i++) {

100  check\_finite(function, mu\_vec[i], "Location parameter", &lp);

101  check\_not\_nan(function, y\_vec[i], "Random variable", &lp);

102  }

103

104  if (size\_y == 0) //y\_vec[0].size() == 0

105  return lp;

106

107  if (include\_summand<propto>::value)

108  lp += NEG\_LOG\_SQRT\_TWO\_PI \* size\_y \* size\_vec;

109

110  if (include\_summand<propto, T\_covar>::value)

111  lp -= 0.5 \* log\_determinant\_ldlt(ldlt\_Sigma) \* size\_vec;

112

113  if (include\_summand<propto,T\_y,T\_loc,T\_covar>::value) {

114  lp\_type sum\_lp\_vec(0.0);

115  for (size\_t i = 0; i < size\_vec; i++) {

116  Eigen::Matrix<typename

117  boost::math::tools::promote\_args<typename scalar\_type<T\_y>::type, typename scalar\_type<T\_loc>::type>::type,

118  Eigen::Dynamic, 1> y\_minus\_mu(size\_y);

119  for (int j = 0; j < size\_y; j++)

120  y\_minus\_mu(j) = y\_vec[i](j)-mu\_vec[i](j);

121  sum\_lp\_vec += trace\_inv\_quad\_form\_ldlt(ldlt\_Sigma,y\_minus\_mu);

122  }

123  lp -= 0.5\*sum\_lp\_vec;

124  }

125  return lp;

126  }

127

128  template <typename T\_y, typename T\_loc, typename T\_covar>

129  inline

130  typename boost::math::tools::promote\_args<typename scalar\_type<T\_y>::type, typename scalar\_type<T\_loc>::type, T\_covar>::type

131  multi\_normal\_log(const T\_y& y,

132  const T\_loc& mu,

133  const Eigen::Matrix<T\_covar,Eigen::Dynamic,Eigen::Dynamic>& Sigma) {

134  return multi\_normal\_log<false>(y,mu,Sigma);

135  }

136

137  template <class RNG>

138  inline Eigen::VectorXd

139  multi\_normal\_rng(const Eigen::Matrix<double,Eigen::Dynamic,1>& mu,

140  const Eigen::Matrix<double,Eigen::Dynamic,Eigen::Dynamic>& S,

141  RNG& rng) {

142  using boost::variate\_generator;

143  using boost::normal\_distribution;

144

145  static const char\* function = "stan::prob::multi\_normal\_rng(%1%)";

146

147  using stan::math::check\_finite;

148  using stan::math::check\_positive;

149  using stan::math::check\_symmetric;

150

151  check\_positive(function, S.rows(), "Covariance matrix rows", (double\*)0);

152  check\_symmetric(function, S, "Covariance matrix", (double\*)0);

153  check\_finite(function, mu, "Location parameter", (double\*)0);

154

155  variate\_generator<RNG&, normal\_distribution<> >

156  std\_normal\_rng(rng, normal\_distribution<>(0,1));

157

158  Eigen::VectorXd z(S.cols());

159  for(int i = 0; i < S.cols(); i++)

160  z(i) = std\_normal\_rng();

161

162  return mu + S.llt().matrixL() \* z;

163  }

164  }

165 }

166

167 #endif

matrix.hpp

stan::max\_size\_mvt

size\_t max\_size\_mvt(const T1 &x1, const T2 &x2)

**Definition:** traits.hpp:575

traits.hpp

check\_size\_match.hpp

stan::math::check\_size\_match

bool check\_size\_match(const char \*function, T\_size1 i, const char \*name\_i, T\_size2 j, const char \*name\_j, T\_result \*result)

**Definition:** check\_size\_match.hpp:13

error\_handling.hpp

stan::math::check\_finite

bool check\_finite(const char \*function, const T\_y &y, const char \*name, T\_result \*result)

Checks if the variable y is finite.

**Definition:** check\_finite.hpp:52

stan::scalar\_type::type

scalar\_type\_helper< is\_vector< T >::value, T >::type type

**Definition:** traits.hpp:139

constants.hpp

check\_symmetric.hpp

traits.hpp

stan::math::check\_symmetric

bool check\_symmetric(const char \*function, const Eigen::Matrix< T\_y, Eigen::Dynamic, Eigen::Dynamic > &y, const char \*name, T\_result \*result)

Return true if the specified matrix is symmetric.

**Definition:** check\_symmetric.hpp:31

stan::math::check\_ldlt\_factor

bool check\_ldlt\_factor(const char \*function, stan::math::LDLT\_factor< T, R, C > &A, const char \*name, T\_result \*result)

Return true if the underlying matrix is positive definite.

**Definition:** check\_ldlt\_factor.hpp:24

check\_ldlt\_factor.hpp

stan::prob::include\_summand

Template metaprogram to calculate whether a summand needs to be included in a proportional (log) prob...

**Definition:** traits.hpp:35

rev.hpp

stan::math::LDLT\_factor

**Definition:** LDLT\_factor.hpp:16

log\_determinant\_ldlt.hpp

stan::length\_mvt

size\_t length\_mvt(const T &)

**Definition:** traits.hpp:561

stan::math::check\_positive

bool check\_positive(const char \*function, const T\_y &y, const char \*name, T\_result \*result)

**Definition:** check\_positive.hpp:54

stan::prob::multi\_normal\_rng

Eigen::VectorXd multi\_normal\_rng(const Eigen::Matrix< double, Eigen::Dynamic, 1 > &mu, const Eigen::Matrix< double, Eigen::Dynamic, Eigen::Dynamic > &S, RNG &rng)

**Definition:** multi\_normal.hpp:139

trace\_inv\_quad\_form\_ldlt.hpp

stan::math::check\_not\_nan

bool check\_not\_nan(const char \*function, const T\_y &y, const char \*name, T\_result \*result)

Checks if the variable y is nan.

**Definition:** check\_not\_nan.hpp:56

stan::agrad::log\_determinant\_ldlt

var log\_determinant\_ldlt(stan::math::LDLT\_factor< var, R, C > &A)

**Definition:** log\_determinant\_ldlt.hpp:48

check\_finite.hpp

size\_

size\_t size\_

**Definition:** dot\_self.hpp:18

stan::VectorViewMvt

**Definition:** traits.hpp:514

stan::prob::multi\_normal\_log

boost::math::tools::promote\_args< typename scalar\_type< T\_y >::type, typename scalar\_type< T\_loc >::type, T\_covar >::type multi\_normal\_log(const T\_y &y, const T\_loc &mu, const Eigen::Matrix< T\_covar, Eigen::Dynamic, Eigen::Dynamic > &Sigma)

**Definition:** multi\_normal.hpp:27

stan::agrad::trace\_inv\_quad\_form\_ldlt

boost::enable\_if\_c< boost::is\_same< T2, var >::value||boost::is\_same< T3, var >::value, var >::type trace\_inv\_quad\_form\_ldlt(const stan::math::LDLT\_factor< T2, R2, C2 > &A, const Eigen::Matrix< T3, R3, C3 > &B)

Compute the trace of an inverse quadratic form.

**Definition:** trace\_inv\_quad\_form\_ldlt.hpp:162


---

[ Stan Home Page ]

*© 2011–2014,
Stan Development Team.*
